# Preparation and evaluation of a lateral flow immunochromatographic nanogold diagnostic kit for brucellosis in sheep

**DOI:** 10.14202/vetworld.2022.2658-2664

**Published:** 2022-11-21

**Authors:** Zainab Mohammed Aboelqassem, Hazem Mohammed Ibrahim, Rafik Hamed Sayed, Hassan Mohamed Sobhy, Sahar Hussein Abdalla Hekal

**Affiliations:** 1 General Organization for Veterinary Services (GOVS), Naddi Alsaeed St. Dokki, Giza Governorate, Giza, Egypt; 2 Veterinary Serum and Vaccine Research Institute, Agricultural Research Center, Giza, Egypt; 3 Central Laboratory for evaluation of Veterinary Biologics, Agricultural Research Center, Giza, Egypt; 4 Department of Natural Resources, Faculty of African Postgraduate Studies, Cairo University, Giza, Egypt

**Keywords:** brucellosis, diagnosis, enzyme-linked immunosorbent assay, lateral flow assay, lateral flow immunochromatographic test, Rose Bengal plate test

## Abstract

**Background and Aim:**

Brucellosis is a zoonotic disease with a worldwide distribution. It has a serious impact on the health of humans and animals, along with a negative impact on the economy. This study aimed to prepare and evaluate the diagnostic performance of a lateral flow immunochromatographic test (LFIT) nanogold diagnostic kit for detecting brucellosis in sheep.

**Materials and Methods:**

A rapidly developed LFIT, in which lipopolysaccharide conjugates with nanogold molecules, was placed on the conjugate pad. One hundred ovine serum samples were tested to detect *Brucella* antibodies (Ab) using the prepared lateral flow immunochromatography assay (LFA) kit and Rose Bengal test. The evaluation of specificity, sensitivity, and accuracy for LFIT and Rose Bengal plate test was conducted using the P04310-10 IDEXX brucellosis ovine/caprine Ab enzyme-linked immunosorbent assay (ELISA) test (gold standard).

**Results:**

The lower amount of *Brucella* Ab in the ovine serum samples was detected and was 1.58 S/P ratio ELISA titer/100 μL using LFIT and with Rose Bengal to detect 1.86 S/P ratio ELISA. The results showed that the developed LFIT had high specificity with no cross-reactivity with other tested bacteria. The calculated sensitivity, specificity, and accuracy of LFIT and Rose Bengal test using the P04310-10 IDEXX brucellosis ovine/caprine Ab ELISA test (gold standard) were 74% and 89%, 81% and 59%, and 76.9% and 66%, respectively.

**Conclusion:**

The present results showed interesting results implying that the LFIA strip test could be used as a substantial diagnostic tool for field screening ovine *Brucella* as an essential step in the control of brucellosis. However, further studies for the validation of the present findings are necessary.

## Introduction

*Brucellosis* is a neglected and highly contagious zoonotic disease known as Malta fever, with a significant socioeconomic concern [1]. It is caused by intracellular and Gram-negative multiple species of the genus *Brucella* that affect a wide host range, including humans who acquire the infection through the consumption of raw milk, animal products, and undercooked meat, or through direct contact with the diseased animals [2]. Each species of *Brucella* has some host preferences, where the most common species that affect human are *B. Melitensis* of small ruminants, *B. Abortus* of large ruminants and *B. suis* of swine [3]. However, despite the fact that the reservoir hosts may be developed due to an infection in non-preferred hosts [4], the disease has a serious negative impact on farm animals. Concerning sheep, the disease causes great economic losses due to its negative effects on reproductive performance, including abortion, decreased fertility, and reduced milk yield [5].

There are several challenges in the control of brucellosis [6]. First, there is a risk of spillover of the infection to wildlife in African countries and the subsequent sustainability of the disease [7]. The control of this sustainable and epidemic infection warrants the development of diagnostic tests that can overcome the defaults of conventional methods, including the need for advanced equipment and professional technicians, which are not available in several developing African countries [8]. Low sensitivity and specificity are the other shortages of traditional methods, such as the Rose Bengal, which may give inaccurate results [9]. One of the rapid and reliable diagnostic tests is the lateral flow immunochromatography assay (LFA) directed toward the detection of immunoglobulin (Ig) M-and IgG-specific antibodies (Ab) against lipopolysaccharide (LPS) [10]. The LFA test has several advantages as it does not require special expertise or advanced equipment, making it more suitable for developing countries [11]. Regarding the improvement of the LFIA sensitivity, the use of Gold Nanoparticles (GNPs) can achieve that, as GNPs carry several advantages, including an easy and well-developed preparation method, stabile optical response, and, importantly, maintaining the Abs’ affinity during conjugation [12].

Therefore, this study aimed to prepare and evaluate the diagnostic performance of a lateral flow immunochromatographic test (LFIT) nanogold diagnostic kit for detecting *brucellosis* in sheep.

## Materials and Methods

### Ethical approval

As per Committee for the Purpose of Control and Supervision of Experiments on Animals guidelines, the approval of the Institute Animal Ethics Committee was not required as the study involving clinical samples and no invasive technique was used.

### Study period and location

The study was conducted from May 2021 to May 2022. The samples were collected from different government veterinary clinics in Giza, Egypt. All laboratory work was done in Veterinary Serum and Vaccine Research Institute.

### Population study

The clinical samples were used to evaluate the prepared LFA strip. A total of 100 ovine serum samples were collected randomly from different cases admitted to different governmental veterinary clinics in Giza, Egypt and tested with the Rose Bengal-colored antigen. The LFIT was prepared and the results were compared with the P04310-10 IDEXX brucellosis ovine/caprine Ab enzyme-linked immunosorbent assay (ELISA) test (gold standard test).

### Gold standard test (P04310-10 IDEXX brucellosis ovine/caprine Ab ELISA test)

The positive standard antisera of *B. melitensis* were obtained from IDEXX brucellosis ovine/caprine Ab test ELISA (cat P04310-10). Ammonium sulfate was used to purify the positive control, and a two-fold serial dilution was used as the positive control. The obtained dilutions were tested by ELISA (100 μL) and LFIT (100 μL). The ELISA was performed according to the instructions in the kit manual. The S/P ratio positive cutoff was 1.20 and that for the suspect cutoff was 1.1.

### Bacterial strain and growth conditions

Reference *Brucella* strain S99 was obtained from the Vaccine and the Serum Research Veterinary Institute and cultured on *Brucella* agar culture medium in 40 plates for an incubation period of 48 h at 37°C. Next, 3–5 mL of *Brucella* broth was taken in an inoculation needle and used for washing the bacteria from the plates’ surface, followed by washing in a sterile glass container and collection of the bacteria in a liquid broth. The bacterial suspension was lysed by heating for 30 min in a water bath at 80°C after checking the broth’s purity and homogeneity [13]. Then, to ensure the killing of bacteria, the culture medium of *Brucella* agar was used for culturing the liquid containing the bacteria and then transferred under sterile conditions in a 250-mL bucket of twistable caps, followed by centrifugation at 4°C for 30 min at 12000× *g*. Then, the bacterial strains’ sediments were placed in sterile tubes.

### Extraction of LPS: Optimized hot phenol method [14]

The hot phenol method was used, albeit with some modifications. Briefly, the LPS of the bacteria was extracted. Then, 30, 50, 75, and 100 mg of wet bacteria were dissolved completely in distilled water (510 μL), followed by heating the obtained cellular suspension to 68°C. Next, we added 570 μL of preheated 90% phenol to the suspension and stirred it for 30 min at the same temperature. Next, the temperature of the sample was rapidly dropped to 5°C in an ice bath, followed by centrifugation at 4°C for 15 min at 8000× *g*. Subsequently, the following four phases were obtained: The top phase contained the phenol-saturated aqueous; the second layer contained white sediment between the top phase and the aqua phase; the third phase contained the aqua-saturated phenol, and the last phase contained the sediment in the lower part of the tube. The first aqueous and the second sediment phases were separated and carefully removed. Then, the phenol phases were carefully separated; for deposition of the nucleic acids and proteins, we added a half volume of cold methanol to it (unlike most bacteria, LPS enters the phenol phase in *Brucella*) and left the samples at 4°C for 30 min and then centrifuged them at 4°C at 1500× *g* for 10 min. Next, the top phenol phase was separated, followed by discarding the protein and nucleic acid sediments and the addition of 50 mg/mL of HCL and stirring for 15 min at 56°C. The mixture was then centrifuged at 1500× *g* at 4°C for 10 min, the supernatant was collected, and the remaining protein sediment was discarded. To obtain the LPS, we added a methanol reagent in three volumes (99 volumes of methanol + 1 volume of sodium acetate-saturated methanol) and stirred the solution for 1 h on an ice bath. Through centrifugation at 8000× *g* for 20 min at 4°C, LPS was deposited and then collected. For the elimination of the remaining phenol, the precipitated LSP was dissolved in 25 μL of distilled water to which 75 μL of cold methanol reagent was added, and the solution was vortexed for 60 min at 27°C and then centrifuged at 8000× *g* for 20 min at 4°C. Then, the pellet was dissolved in 500 μL of distilled water and the sediment concentration was quantified by a spectrophotometer (Thermo Fisher Scientific, USA).

### Preparation of Ab against LPS in rabbits (loaded in the control line)

The same volume of both LPS of *Brucella* and complete Freund’s adjuvant were mixed [15]. First, male rabbits were injected intradermally with the emulsion at a dose of 0.5 mg/kg. The preimmunized rabbits were injected S/C with booster doses of a mixture of antigen and an oily incomplete Freund’s adjuvant at a dose of 0.15 mg/kg at intervals of 2 weeks for 8 weeks. After 10 days of the last injection, the serum was collected and the rabbit polyclonal antibody specific against the LPS antigen was obtained.

### Purification of Igs from rabbit serum

Purification of Igs from rabbit polyclonal Ab was performed with caprylic acid as described by Bergmann-Leitner *et al*. [16]. After centrifugation of 25 μL of each serum at 10000× *g* for 20–30 min, the pellet was discarded. Then, 50 mL of 0.06 M sodium acetate buffer (pH 4.6) was added and the solution was mixed in a beaker on a magnetic stirrer. During the stirring at 25°C, drop by drop of caprylic acid (2.02 mL) was added for 30 min and the mixture was centrifuged at 10,000× *g* for 20 min. Next, the supernatant was obtained and the pellet was discarded. The obtained supernatant was dialyzed against phosphate-buffered saline (PBS) buffer at 4°C overnight with three buffer changes. Finally, a spectrophotometer (Thermo Fisher Scientific) was used to measure the concentration of purified IgG.

### Preparation of LPS conjugated with 40-nm nanogold (loaded in the conjugation pad)

#### Preparation of colloidal gold 40-nm nanoparticles

Nanoparticles were prepared as described by Singh *et al*. [17]. CG nanoparticles were adjusted to 40-nm diameter size and then boiled with vigorous stirring on purified water (50 mL) with 0.01% (w/v) sodium citrate. To this, we added 1 mL of 1% HAuCl4 rapidly. After 2 min, when the color of the solution turned red, another 10 min of boiling was applied. Finally, 0.02% (w/v) of sodium azide was added. After cooling, a spectrophotometer (Thermo Fisher Scientific) was used to check the diameter of the obtained nanoparticles within the range of 400–600 nm.

#### Conjugation of LPS with nanogold [18]

For this procedure, 25 μL of 1% LPS solution was added to a solution of colloidal gold at a volume of 125 μL. Then, the mixture was incubated for 15 min at 27°C and then treated with 10% NaCl solution (100 μL). The color of the samples changed from wine red to blue with a decreasing antigen concentration. The required antigen concentration for labeling colloidal gold was the lowest antigen concentration that did not change the color. The optical antigen solution was equal to 200 μL. The solution was gently mixed for 10 min, and then polyethylene glycol (20,000, 1% [m/v] final concentration) was used for blocking with stirring for another 15 min and centrifugation at 10,000× *g* for 30 min. The obtained gold pellets were suspended in 1 mL of the dilution buffer (20 mM Tris/HCI buffer (pH 8.2) containing 3% [w/v] sucrose 1% [w/v] bovine serum albumin, and 0.02% (w/v) sodium azide) and stored at 4°C until further use.

#### Purchase of donkey anti-ovine Ab

Donkey polyclonal Ab against sheep/goat IgG was purchased from BioRad, USA (product no. STAR88A).

#### Preparation of LFIT [18]

Sample pad

Phosphate-buffered saline at pH 7.2 was used to saturate glass fiber. The solution contained 0.5% (w/v) triton X1000 and 3% Tween-20. Then, the fiber was dried at 37°C and maintained at room temperature (30°C) in a dry place until use.

The conjugate pad

We treated the glass fiber with 0.1% Tween-20 for 10 min with drying at 60°C and cut it into sections of sizes 30 × 0.5 cm, followed by saturating it with 0.15 mL of LPS-conjugated nanogold. Finally, the fiber was dried at 37°C for 1 h and stored under dry conditions until use.

Nitrocellulose (NC) membrane

**Figure-1:**
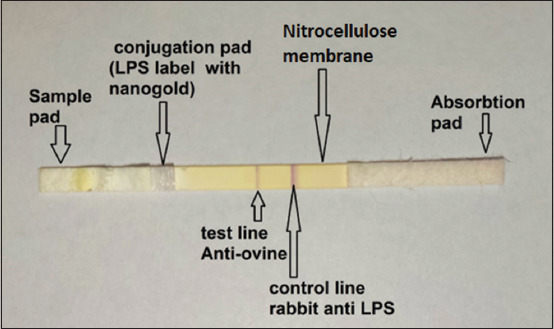
Lateral flow immunochromatographic test.

Two lines were applied on the NC membrane (25 × 300 mm) using a dispenser (Iso flow USA). Then, 1.5 mg/0.1 mL of the rabbit anti-ovine Ab was distributed near the bottom as the test line (1 μL/cm line), while the rabbit antibody against LPS antigen (1 mg/mL) was placed at the upper portion as the control line (1 μL/cm line). The two lines were at a distance of 6 mm from each other. The membrane was dried at room temperature (30°C) for 2–6 h after applying the test line. Then, the membrane was immersed in a blocking buffer to block it. When the membrane became completely wet, it was immersed 5 times in the first PBS for washing, followed by another 5 rounds in the second PBS solution. Finally, the top laminate was used to cover the membrane and then cut into test strips of width 0.5 cm using an automated cutter machine as shown in Figure-1.

Interpretation of the test

Two red bands at the test and control zones appeared with no further addition of the reagent. If the tested samples of ovine serum contained low Ab concentration against *Brucella* than the detection limit, only one band could be visualized in the control zone. Otherwise, if no band was seen at the control and test zones, it indicated the invalidity of the test. The concentration of Ab against *Brucella* or LPS in the tested serum samples controlled the intensity of the test line in direct proportion. The control zone served as the positive control to confirm the migration of functional, conjugated antigens in the system. The total time required for the test was <5 min. The estimation of the results of the test strip could be performed either visually or with the naked eye.

## Results

### Sensitivity test

**Figure-2:**
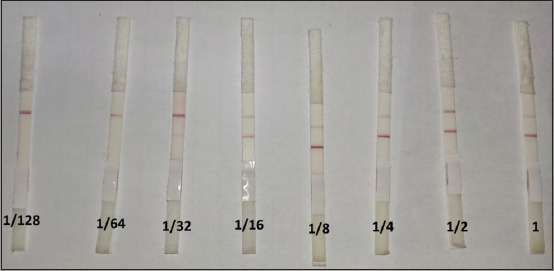
The sensitivity test of lateral flow immunochromatographic test using positive standard antisera of *Brucella melitensis* diluted with 2-fold serial dilution.

A lower amount of Ab against *Brucella* was detected in the serum sample (1.58 S/P ratio ELISA titer/100 μL) using LFIT. The Rose Bengal test result was a 1.86 S/P ratio ELISA, as shown in Figure-2 and Table-1.

**Table-1:** The sensitivity test of LFIT, ELISA, and Rose Bengal test using positive standard antisera of Brucella melitensis diluted with a two-fold serial dilution.

Dilution	1	1/2	1/4	1/8	1/16	1/32	1/64	1/128
ELISA S/p ratio	2.21 positive	2.04 positive	1.98 positive	1.86 positive	1.58 positive	1.43 positive	1.29 positive	1.15 suspect
LFIT	+	+	+	+	+	+/-	-	-
Rose Bengal test	+++	+++	++	+	-	-	-	-

LFIT = Lateral flow immunochromatographic test, ELISA = Enzyme-linked immunosorbent assay

### Specificity test

**Figure-3:**
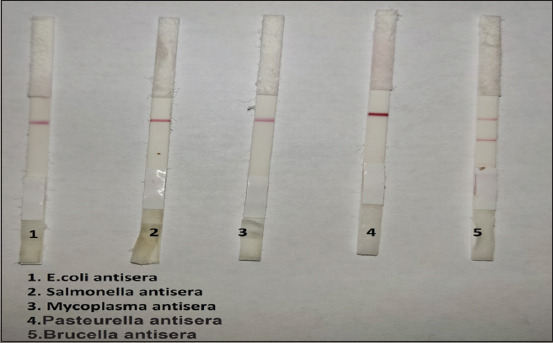
Specificity test of the diagnostic kit using standard anti-sera of *Salmonella*, *Escherichia coli*, *Mycoplasma*, *Pasteurella* antisera, and positive *Brucella*.

Standard antisera of *Salmonella*, *Escherichia coli*, *Mycoplasma*, *Pasteurella* antisera, and positive *Brucella* were tested with the prepared kit. The standard antisera of *Salmonella*, *E. coli*, *Mycoplasma*, and *Pasteurella* antisera gave negative results. But positive *Brucella* antisera gave a positive result (Figure-3).

### Determination of specificity, sensitivity, and accuracy for LFIT using P04310-10 IDEXX brucellosis ovine/caprine Ab ELISA test (gold standard)

#### Validity test

Validation test was based on statistical analysis and evaluation of LFIT.

#### Sensitivity (true positive rate)

Indicated the ability of diagnostic kits to correctly and accurately identify the percentage of the sample containing *Brucella* Ab:


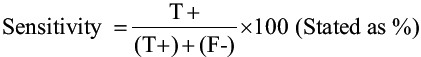


#### Specificity (True negative rate)

Indicated the ability of the diagnostic kits to correctly and accurately identify the percentage of the sample not containing *Brucella* Ab:

#### Accuracy (validity)

It demonstrated how much the measurement represented the true situation of what was being measured.


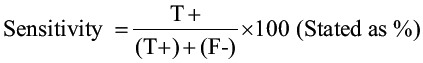


**Figure-4:**
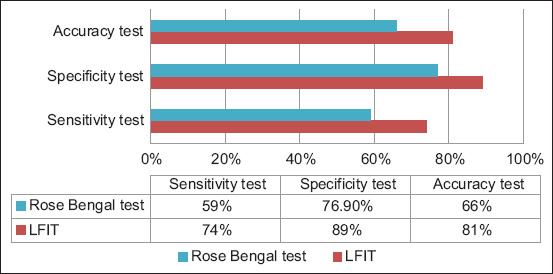
Evaluation of diagnostic accuracy, sensitivity, and specificity of lateral flow immunochromatography assay, Rose Bengal test using P04310-10 IDEXX brucellosis ovine/caprine antibodies enzyme-linked immunosorbent assay test (gold standard).

The results of LFIT and Rose Bengal compared with ELISA were calculated at (T+), (F+), (F−) and (T−) and found to be 40, 5, 14, and 41 and 36, 9, 25, and 30, respectively. The sensitivity, specificity, and accuracy of LFIT and Rose Bengal test, when compared to ELISA, were calculated and found to be 74%, 89%, and 81% and 59%, 76.9%, and 66%, respectively, as shown in Table-2 and Figure-4.

**Table-2:** Evaluation of the diagnostic kit (Validity test) using P04310-10 IDEXX brucellosis ovine/caprine Ab ELISA test (gold standard).

LFIT and Rose Bengal test	ELISA			Sensitivity test	Specificity test	Accuracy test

	+ ve	- ve	total			
LFIT						
+ve	40 (T+)	5(F+)	45	74%	89%	81%
-ve	14 (F-)	41(T-)	55			
total	54	46	100			
Rose bengal test						
+ve	36 (T+)	9(F+)	45	59%	76.9%	66%
-ve	25(F-)	30(T-)	55			
total	61	39	100			

Gold Standard = The means by which one can detect *Brucella* Ab whether it is truly present or not. In this study, ELISA was the gold standard, False positive (F+) = is when the diagnostic kits indicate that the sample contains bacteria, but in fact it does not contain this *Brucella* Ab, False negative (F-) = is when the diagnostic kits indicate that the sample does not contain bacteria, but in fact it contains the *Brucella* Ab, True positive (T+) = is when the diagnostic kits indicate that the sample contains bacteria and indeed contains the *Brucella* Ab, True negative (T-) = is when the diagnostic kits indicate that the sample free from *Brucella* Ab and indeed it is free, LFIT = Lateral flow immunochromatographic test

## Discussion

Accurate and rapid diagnosis of re-emerging zoonotic diseases is critical for their control and monitoring. In brucellosis, the early diagnosis of the infected animals is necessary for controlling and eradicating the disease [19]. Therefore, the availability of a rapid and simple test that does not require highly sophisticated equipment and laboratories and that can be used as a screening field test is of great importance [20]. Lateral flow immunochromatography assay is a rapid immunofiltration test based on the conjugation of Ab to colloidal nanogold [21]. This labeling molecule has a high affinity for proteins and biomolecules, which enhances their stability and allows specific optical signaling [22]. Hence, colloidal nanogold remains the most dominant colorimetric label [23]. This study indicates that LFIA can be used as a substitution screening test for brucellosis. The developed LFA strips could detect the Ab against *Brucella* in a serum sample at a minimal concentration relative to the Rose Bengal test, where the minimal amount of Ab against *Brucella* in the serum sample detected was 1.51.58 and 1.86 S/P in the LFA strips and Rose Bengal test, respectively; these lower cutoff values indicate a high sensitivity, which is one of the most important features of a good test [24]. In other studies on the detection of *Corynebacterium pseudotuberculosis*, the minimal concentration that gave positive results in LFIT was CFU/0.1 mL [25], while for, *Staphylococcus aureus*, the sensitivity was 106 CFU/0.1 mL [26]. For the evaluation of the specificity level, the standard antisera of *Salmonella*, *E. coli*, *Mycoplasma*, *Pasteurella* antisera, and positive *Brucella* were tested with the prepared kit. The only positive result obtained using the *Brucella* antisera declared the good specificity level of the developed kit, and no cross-reactivity was detected with other tested bacteria. The evaluation of the relative specificity, sensitivity, and accuracy for LFIT using P04310-10 IDEXX brucellosis ovine/caprine Ab ELISA test (gold standard) was performed, and the results of sensitivity, specificity, and accuracy of LFIT and Rose Bengal test were compared with those of ELISA and found to be 74%, 89%, and 81% and 59%, 76.9%, and 66%, respectively. A nearly similar result was obtained by Elyazeed *et al*. [27], who found that the LFIT had low sensitivity with high specificity (77.5% and 92%), respectively, for the detection of *Mycoplasma gallisepticum*. For the detection of brucellosis, the sensitivity and specificity were recorded as 78.57% and 93.07%, respectively [28]. The results of other study using LFA for the detection of brucellosis have demonstrated sensitivity and specificity of 87.1% and 92.6%, respectively [29]. The LFIT showed sensitivity, specificity, and accuracy of 91%, 80%, and 90%, respectively, for the detection of *Salmonella* enteritidis in poultry [30].

## Conclusion

It can be concluded that the LFIA strip test can be used as a substantial diagnostic tool for field screening of ovine *Brucella* as an essential step toward the control of brucellosis. However, further studies are warranted for confirmation.

## Authors’ Contributions

ZMA: Collection of the samples and the laboratory work. HMI, RHS, and SHAH: Experimental design, laboratory work, data analysis, and drafted and revised the manuscript. HMS: Supervised the study. All authors have read and approved the final manuscript.
